# Protein–lipid interplay at the neuromuscular junction

**DOI:** 10.1093/jmicro/dfab023

**Published:** 2022-02-18

**Authors:** Nigel Unwin

**Affiliations:** MRC Laboratory of Molecular Biology, Francis Crick Avenue, Cambridge Biomedical Campus, Cambridge, Cambridgeshire CB2 0QH, UK

**Keywords:** nicotinic acetylcholine receptor, cholesterol microdomain, postsynaptic membrane, synaptic transmission, cryo-EM

## Abstract

Many new structures of membrane proteins have been determined over the last decade, yet the nature of protein–lipid interplay has received scant attention. The postsynaptic membrane of the neuromuscular junction and *Torpedo* electrocytes has a regular architecture, opening an opportunity to illuminate how proteins and lipids act together in a native membrane setting. Cryo electron microscopy (Cryo-EM) images show that cholesterol segregates preferentially around the constituent ion channel, the nicotinic acetylcholine receptor, interacting with specific sites in both leaflets of the bilayer. In addition to maintaining the transmembrane α-helical architecture, cholesterol forms microdomains – bridges of rigid sterol groups that link one channel to the next. This article discusses the whole protein–lipid organization of the cholinergic postsynaptic membrane, its physiological implications and how the observed details relate to our current concept of the membrane structure. I suggest that cooperative interactions, facilitated by the regular protein–lipid arrangement, help to spread channel activation into regions distant from the sites of neurotransmitter release, thereby enhancing the postsynaptic response.

## Introduction

The 1972 ‘fluid mosaic model’ of membrane structure [1] envisioned membrane proteins as single molecules or complexes that were embedded within a fluid lipid bilayer but otherwise similar to their counterparts in solution. The protein chains integrated stably by folding with their polar and nonpolar parts partitioned according to whether they faced solvent or the hydrophobic portions of the lipids. Lipids in general were considered to interact rather non-specifically with the protein surfaces, providing stability like solvent in the water-exposed parts. While structural studies of a wide range of membrane proteins have since demonstrated the overall validity of this concept, the structural properties and molecular organization of the lipids have received less scrutiny.

Yet far from being passive partners, lipids are often found to play vital roles in enabling, optimizing and coordinating protein function. Some proteins incorporate sites for longer lived interactions and a higher degree of specificity in terms of preferred lipid species, which may act as a ‘co-factors’ essential to the biological mechanism. Depending on the local membrane composition, the lipids themselves may also create more ordered and less fluid regions (‘lipid rafts’) within the bilayer, which too can influence how a protein works. The fact that natural bilayers contain lipid hydrocarbon chains with great heterogeneity in terms of length, saturation and headgroup composition, adds a further layer of complexity. Clearly, for a proper understanding of any particular cell membrane, it is important not to rely on simplified model systems but to view the protein–lipid and lipid–lipid interplay as they exist *in situ*.

**Fig. 1.**
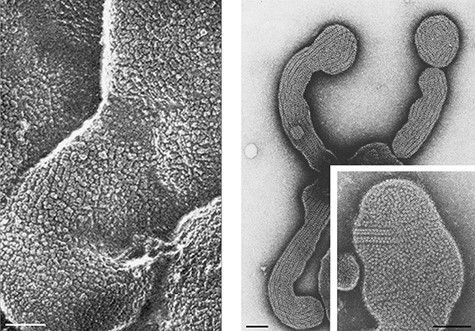
Organization of acetylcholine receptors in postsynaptic membranes from frog neuromuscular junction (freeze-fracture, deep-etch image of *in situ* synaptic surface, left) and *Torpedo* electric organ (negative-stain image of extracted membrane, right). Both images show long, close-packed ribbons of receptors extending across the membrane surfaces. The enlargement of an isolated vesicle on the right shows that ribbons are comprised of dimers of receptors in oblique alignment (see also Fig. 2b). While the structural findings reported here have been performed entirely on the more experimentally tractable *Torpedo* membrane, the close architectural similarities between the two systems, and their shared developmental origin, make it unlikely that their protein–lipid interactions and organization are fundamentally different. The images are from Hirokawa and Heuser [4] (left) and Brisson and Unwin [5] (right). Scale bar, 1000 Å.

The cholinergic postsynaptic membrane of the neuromuscular junction and *Torpedo* electrocytes provides a unique opportunity to visualize and characterize how protein and lipid components act together in an intact physiological setting. This specialized region of the muscle (or electrocyte) cell membrane comprises a cholesterol-rich phospholipid bilayer, most densely populated by a single membrane-spanning protein, the nicotinic acetylcholine receptor: a transmitter-gated ion channel (for recent reviews, see Refs. [2,3]). The receptors pack tightly next to one another in the lipid matrix and form long dimer ribbons across the membrane surfaces ( [4–6]; Fig. 1). Here I bring to centre stage the protein–lipid interplay underpinning these channel arrays and suggest how it may help to achieve the properties of the postsynaptic membrane as a finely tuned mediator of fast synaptic transmission.

**Fig. 2.**
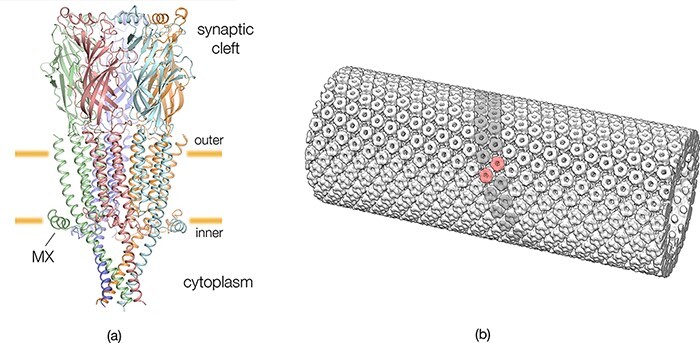
Structures of the acetylcholine receptor and a tubular vesicle from *Torpedo* postsynaptic membrane. (a) The receptor is a heteropentamer (stoichiometry: α_δ_, α_γ_, β, γ, δ), which includes four TM helices (M1–M4) in each subunit, and a transverse sub-membrane helix, MX. The structure shown is that of a recent model (PDB ID 6uwz [9]), modified such that its transmembrane portion fits the densities of the (cholesterol-complexed) receptor in the tubes (Unwin, unpublished). The orange bars identify the levels of the phosphate moieties of the outer and inner phospholipid headgroups, which are 30 Å apart [7]; subunit colours: α_γ_, red; α_δ_, orange; β, green; γ, cyan; δ, blue. (b) The tubular vesicle consists of curved ribbons of δ subunit-linked receptor dimers packed closely together and embedded in a bilayer matrix, which is composed of the native lipids (one ribbon, grey; one dimer, pink).

## The postsynaptic membrane

Electron microscopical technology has undergone remarkable advances since freeze-fracture and negative-staining techniques first revealed the specialized membrane organization shown in Fig. 1. It is now routine to apply cryo-methods, in which rapid freezing of the sample in solution, together with use of a cold stage, allows one to both capture and image a specimen as it would be in its normal physiological setting. Although the signal-to-noise ratio obtainable from direct imaging is limited by radiation damage, this deficiency is surmountable by combining and averaging different low-dose views of identical copies of the same molecules.

Molecular averaging can be performed readily on postsynaptic membranes from *Torpedo* by exploiting the fact that, when isolated, they form tubular vesicles having the receptors arranged on a helical surface lattice (Fig. 2). This lattice is built from closely packed ribbons of receptor dimers embedded in their natural cholesterol-rich phospholipid bilayer matrix [7,8] and has a range of curvature similar to that at the crests of the junctional folds. In other words, the tubular vesicles recapitulate near perfectly the *in situ* synaptic organization and at the same time facilitate image averaging through imposition of the helical symmetry.

**Fig. 3.**
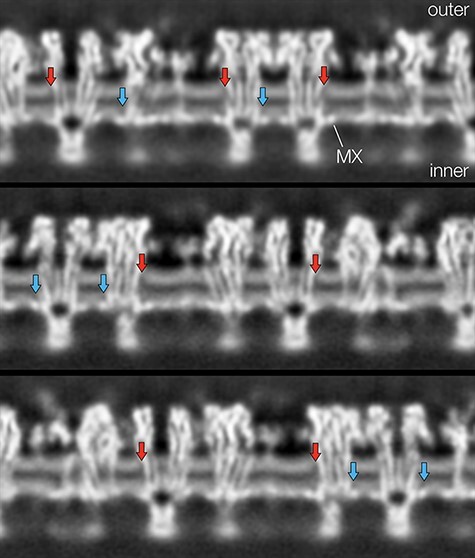
Cross-sections through a helical reconstruction displaying the postsynaptic membrane in profile. Cholesterol in the outer leaflet gives rise to gaps next to the protein surfaces (red arrows) in the otherwise continuous (light grey) densities associated with the phospholipid headgroups. A similar weakening of density associated with cholesterol occurs in the inner leaflet, above the MX helices (blue arrows).

Figure 3 shows cross-sections through the postsynaptic membrane obtained from the cryo-images after helical reconstruction [7]. These views are dominated by paired tracks of density corresponding to the strong electron-scattering phospholipid headgroups and by irregular blocks of density corresponding to individual receptors. The headgroups on either side of the bilayer, measured from their peak densities, are 30 Å apart. The ‘lines’ crossing the lipid bilayer and ‘dots’ at their bases can be identified, respectively, with the TM helices and MX in Fig. 2a. The lines (i.e. TM helices) extend slightly beyond the outer phospholipid headgroups before joining with the ligand-binding domain in the synaptic cleft.

One notable feature of the outer leaflet of the bilayer is a weakening of densities next to some of the TM helices, interrupting the otherwise rather uniform dense band associated with the large phospholipid headgroups (red arrows, Fig. 3). This is due to the presence of cholesterol [7,8], which makes up 40–50 mol% of the membrane lipids [2]. Unlike the phospholipids, cholesterol exposes no sizeable headgroup (only a hydroxyl) and so contributes no mass this far from the hydrophobic core. A similar weakening of density, also attributable to cholesterol, is present above the MX helices in inner leaflet of the bilayer (blue arrows, Fig. 3).

## Cholesterol around the receptor

**Fig. 4.**
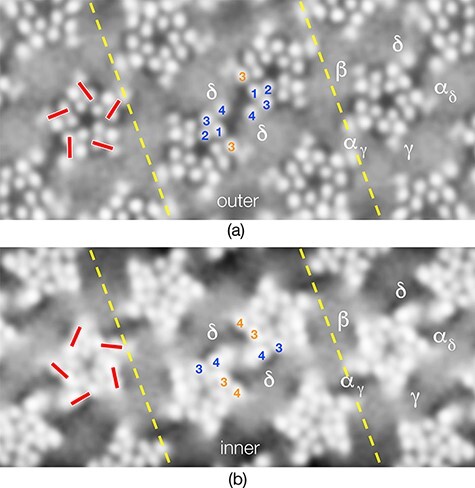
Organization of cholesterol next to acetylcholine receptors, viewed in tangential sections encompassing the phospholipid headgroups. (a) Outer leaflet and (b) inner leaflet: cholesterol-rich patches appear as small gaps (dark grey) among the headgroup densities (smooth light grey areas) next to the TM helices (red bars), and as more extended gaps – microdomains – bridging neighbouring proteins. The pair of receptors comprising the central δ–δ dimer is bridged by microdomains in both leaflets of the bilayer. The pairs of broken lines track a dimer ribbon (seen at lower magnification in Figs. 1 and 2b). The numbering 1–4 identifies TM helices M1–M4. Adapted from [7].

In-plane views encompassing the phospholipid headgroups (Fig. 4) reveal how the cholesterol component is organized around the receptors. As in the cross-sections just described and because of its tiny headgroup, cholesterol creates gaps or small water-filled patches, among the phospholipid-headgroup densities. These patches lie next to each receptor subunit in equivalent regions (red bars). They are on their clockwise faces in the outer leaflet and anti-clockwise faces in the inner leaflet.

Although not yet resolved as discrete entities, the approximate locations of the cholesterol molecules with respect to the TM helices can be deduced by comparing the structure of the membrane-bound receptor with that of the detergent-solubilized receptor [9], where the cholesterol has been extracted. In the solubilized receptor, at the level of the outer phospholipid headgroups, the spacings between TM helices M4–M1 and M1–M3 are contracted, as if cholesterol wedges between them to hold them apart [7]. On the other hand, at the level of the inner phospholipid headgroups, the spacings between the TM helices are unaltered, implying that these cholesterols simply lie against the lipid-exposed surfaces, consistent with the cross-sectional views.

**Fig. 5.**
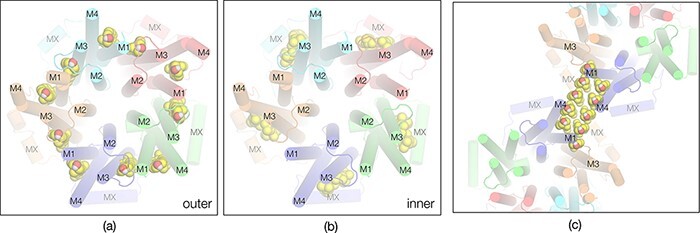
Hypothetical arrangements of cholesterol molecules based on the cryo-EM images from *Torpedo*: (a) wedging between helices M4–M1 and M1–M3 in outer leaflet; (b) supporting helices M3 and M4 in inner leaflet; (c) bridging the δ–δ dimer in the outer leaflet. Subunit colours as in Fig. 2a.

Figure 5a and b show the inferred cholesterol arrangements in the outer and inner leaflets. Although the precise locations of these cholesterols are not yet known, the distinct complexes they form with the receptor at the two levels hint at how this lipid integrates with the transmembrane architecture to achieve full ion channel function. The outer leaflet harbours the gate of the ion channel [7]. Hence, the cholesterol-stabilized splayed-apart arrangement of helices at this level may be needed to create space for the pore-lining M2 helices to move freely between closed and open configurations [10]. The inner leaflet harbours the narrowest portion of the open pore [7]. The stiffness imposed by an encircling ring of rigid sterol groups at this level would limit flexibility and so may help to make ion discrimination more precise.

## Cholesterol microdomains

Most of the cholesterol-interacting regions on the TM helices are associated with only small patches of cholesterol. However, these patches become more extensive, forming microdomains that bridge neighbouring receptors, when brought into close proximity. The microdomains are apparently stabilized by properties of the protein surfaces and by the high cholesterol content of the postsynaptic membrane. The TM helix M4, which has an affinity for cholesterol [11] and tilts into the lipids away from the body of the receptor, is a stabilizing influence on the outer leaflet. The sub-membrane helix MX, which sterically excludes the large phospholipid headgroups from the vicinity of the TM helices, is a stabilizing influence inner leaflet. Two kinds of microdomain exist in the inner leaflet because there are two distinct places where neighbouring cholesterol-interacting sites (on apposed δ/α_δ_ subunits and apposed α_γ_ subunits) both overlie the MX helices and come close to each other.

Interestingly, the central δ–δ dimer is bridged by microdomains in both leaflets of the bilayer, whereas the interface made by neighbouring α_γ_ subunits is bridged by a microdomain only in the inner leaflet. Together the microdomains create an extensive two-dimensional network, involving just δ and α, which connects receptors along lines running obliquely to the vesicle axis. As I discuss later, these lines of ion channels, linked together in alternating orientations, may play a role in communicating gating activity to regions distant from the sites where acetylcholine is released.

## Membrane structure

The lipid bilayer component of the postsynaptic membrane has a fairly uniform thickness with, at most, minor modifications next to the protein surfaces. The 30 Å peak-to-peak separation of the phospholipid headgroups (corresponding to the average separation of the phosphate moieties [12]) may seem unusually small compared with that of other cholesterol-containing membranes [12–16]. For example, X-ray diffraction of myelin, the best studied cell membrane, yields peak-to-peak values of 43–48 Å [15,16]. However, the ∼15 Å discrepancy with myelin probably reflects the fact that about 25% of its hydrocarbon chains are four to five carbon atoms longer than the average chain length in other membranes [17].

Moreover, the headgroup separations of the cholinergic membrane and those mediating parallel functions at central synapses are similar, according to recent cryo-electron tomographic reconstructions [18,19]. A likely explanation, in line with other structural evidence [13,20], is that bilayer thickness is modulated primarily by the properties of the embedded protein, rather than by cholesterol, in regions where the protein is abundant.

The observed partial separation of cholesterol from the lipid matrix to form networks of bridging microdomains may be a common occurrence in cell membrane specializations, where both protein and cholesterol exist at high concentration. Little is yet known how the cholesterol is organized within a microdomain. The sterol assemblies may incorporate some degree of order, as depicted in Fig. 5c, with ring-to-ring packing conferring additional rigidity. They could thereby provide a way of stabilizing transient or long-term communication between proteins and hence function to fine-tune the activity of ion-channel clusters at central synapses. A single cholesterol forms a molecular cross-link between the AMPA receptor and its auxiliary subunit cornichon [21], but cross-links entailing multiple cholesterols are likely to be resolved eventually in other whole-membrane studies.

Are the cholesterol-bridge networks described here and the light microscope-identified regions, often referred as ‘lipid rafts’, essentially the same thing? The presence of the network is consistent with light-microscopical evidence from *Torpedo* membrane fragments [22] and cultured myotubes [23–25] demonstrating that acetylcholine receptors cluster in more ordered cholesterol-rich regions of the cell membrane. Other transmitter-gated ion channels of the central nervous system also reside in cholesterol-rich regions [26,27]. However, lipid rafts are normally considered to be thickened ‘liquid-ordered’ areas of membrane, enriched in both cholesterol and sphingolipids and spanning both leaflets of the bilayer [28].

The features of the cholinergic postsynaptic membrane, now seen at higher resolution, are not fully compatible with this view. Apparently, it is the protein surfaces, not sphingolipids (which are rare in purified *Torpedo* membrane [29]), that play the major part in stabilizing the cholesterol microdomains. Also, the microdomains are arranged differently in the two leaflets and do not overlap significantly, as required to span both leaflets. The network in Fig. 4 most likely would have arisen through a co-assembly mechanism, whereby the cholesterol aggregation is stabilized by the coming together of appropriate protein surfaces, rather than through a mechanism involving recruitment of proteins to pre-existing liquid-ordered regions.

## Fast synaptic transmission

The postsynaptic membrane has a remarkably regular design, suggesting it might function more like a coordinated molecular assembly than simply a crowded grouping of ion channels behaving as independent units. The embedded protein and cholesterol together create a unique microdomain network, which stabilizes the channels in specific orientations and positions relative to one another. The δ subunits of neighbouring receptors make one set of microdomain contacts; the α_γ_ subunits of neighbouring receptors make the other. These two sets of contacts are contiguous, and they bring together structural elements that are important in initiating and terminating the gating movements, i.e. loop C framing the acetylcholine binding site in the α_γ_ subunits [10,30] and the transmembrane portion of the δ subunits [8,31].

The possibility that these microdomain-linked contacts facilitate coordinated activity is consistent with single-channel recordings made on clustered *Torpedo* receptors reconstituted in cholesterol-rich planar membranes [32]. Whereas the monomer channels exhibit the gating behaviour characteristic of single receptors, the observed conductance doubles under conditions where the channels aggregate, becoming equal to the conductance of the native δ–δ dimer. In other words, the monomers are able to function cooperatively and when paired, as in a dimer, give rise to synchronous gating activity. In addition, with even larger clusters, multiple synchronized gating (implicating at least three δ–δ dimers) occurs.

Hence, the structural findings and single-channel evidence concur in supporting the idea that the real ‘functional unit’ may be a small cluster, or linear array, of channels and that activation can propagate by conformational spread [33] within the cluster, without the need for acetylcholine binding to induce individual channel-opening events. Given the context *in situ* of a restricted active zone, where acetylcholine is released, opposite a curved junctional membrane, where some of the reacting channels are less accessible to acetylcholine than others [34–36], the spread in channel opening through a cooperative mechanism may play a significant part in achieving the maximum postsynaptic response.

**Fig. 6.**
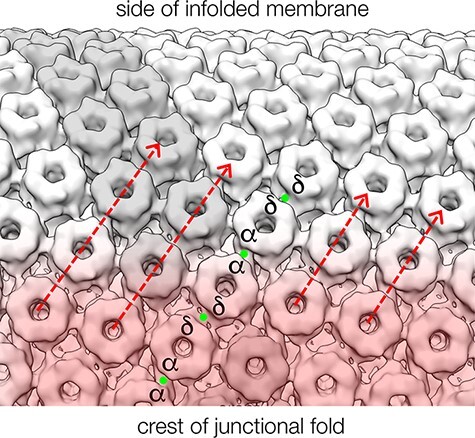
Optimal packing of acetylcholine-receptor channels in the vicinity of the active zone, based on image reconstruction from *Torpedo* tubular vesicles (dimer ribbon in grey; see also Fig. 2b). Channels at the crest of the junctional fold are closest to the active zone, where acetylcholine is released (pink shading). Those located down the side of the infolded membrane (and at more distant regions within the synaptic cleft) are exposed to less neurotransmitter, which is diluted further through its rapid hydrolysis by acetylcholinesterase [37]. Cooperative interactions, implicating the cholesterol-bridged (green dots) δ–δ dimers and α_γ_–α_γ_ contacts, may help to spread channel activation into the side region (arrows) and into distant cleft regions, thereby magnifying the postsynaptic response.

Figure 6 illustrates how the potentially cooperative δ and α_γ_ subunit-linked arrays would extend from the crest of the junctional fold to the side of the infolded membrane, according to the structure of the tubular vesicles from *Torpedo*.

## Conclusion and outlook

The preceding paragraphs underscore the value of imaging the whole postsynaptic membrane, as well as its components separately, to gain a more complete understanding of neuromuscular transmission. The structure of detergent-solubilized acetylcholine receptors in an artificial lipid environment is not the same as it is in the native membrane, complexed with cholesterol. The lipid-microdomain environment at the synapse would be distinct from the more random lipid organization in other parts of the cell membrane. An integrated perspective reveals arrays of ion channels and connecting microdomains assembled in a way that seems designed to promote coordination of ion-channel activity. Spread of channel gating movements through cooperative interactions could have a profound impact on the efficacy of the postsynaptic response.

Similar principles to those discussed are likely to be at work in stabilizing and coordinating the activity of clustered transmitter-gated ion channels at central synapses. Do these channels also engage in cholesterol-modulated coupling to enhance the postsynaptic response? While it may not be possible to exploit ordered arrays to evaluate their protein–lipid interplay, the technique of cryo-electron tomography is advancing rapidly and may soon begin to resolve similar or other relevant whole-membrane details. Complementing this information, additional insight will undoubtedly emerge from single-particle cryo-EM studies of isolated synaptic assemblies, if they can be retained in close-to-cellular membrane settings.
